# Quantitative Model for Ion Transport and Cytoplasm Conductivity of Chinese Hamster Ovary Cells

**DOI:** 10.1038/s41598-018-36127-3

**Published:** 2018-12-13

**Authors:** Azita Fazelkhah, Katrin Braasch, Samaneh Afshar, Elham Salimi, Michael Butler, Greg Bridges, Douglas Thomson

**Affiliations:** 1 Department of Electrical and Computer Engineering, University of Manitoba, Winnipeg, R3T 2N2 Canada; 2 Department of Microbiology, University of Manitoba, Winnipeg, R3T 2N2 Canada; 3 National Institute for Bioprocessing Research and Training, Dublin, Ireland

## Abstract

In mammalian cells cytoplasm ion concentrations and hence cytoplasm conductivity is an important indicator of their physiological state. Changes in the cytoplasm conductivity has been associated with physiological changes such as progression of cancer and apoptosis. In this work, a model that predicts the effects of physiological changes in ion transport on the cytoplasm conductivity of Chinese hamster ovary (CHO) cells is demonstrated. We determined CHO-specific model parameters, Na^+^/K^+^ ATPase pumps and ion channels densities, using a flux assay approach. The obtained sodium (P_Na_), potassium (P_K_) and chloride (P_Cl_) permeability and Na^+^/K^+^ ATPase pump density were estimated to be 5.6 × 10^−8^ cm/s, 5.6 × 10^−8^ cm/s, 3.2 × 10^−7^ cm/s and 2.56 × 10^−11^ mol/cm^2^, respectively. The model was tested by comparing the model predictions with the experimentally determined temporal changes in the cytoplasm conductivity of Na^+^/K^+^ ATPase pump inhibited CHO cells. Cells’ Na^+^/K^+^ ATPase pumps were inhibited using 5 mM Ouabain and the temporal behavior of their cytoplasm conductivity was measured using dielectrophoresis cytometry. The measured results are in close agreement with the model-calculated values. This model will provide insight on the effects of processes such as apoptosis or external media ion concentration on the cytoplasm conductivity of mammalian cells.

## Introduction

Chinese Hamster ovary (CHO) cells are used in the production of 70% of all biopharmaceuticals^1^. They are also extensively employed in medical and biological research studies as they share the characteristics of many mammalian cells. The dynamics of cytoplasm ions behavior is important in CHO cells, as well as other mammalian cells, as a significant portion of cells energy is expended to control the flow of ions across the cell membrane. Changes in ionic content of cells can be an indication of impaired cellular functions and is possible to be detected by measuring the cells cytoplasm conductivity^2,3^. There has been studies showing that cytoplasm conductivity is affected by various processes such as apoptosis^4–6^, progression of cancer^7–9^, differentiation of stem cells^10^, separation of healthy and tumor cells^11^, and drug treatments^9^. Table 1 shows changes in cytoplasm conductivity of various cell lines, as their physiological state changes. In order to link the cytoplasm conductivity of cells to their physiology, there is a need for a quantitative model of ion transport and its relationship with the cytoplasm conductivity. In this study, we develop a quantitative model of ion transport that also estimates cytoplasm conductivity for Chinese hamster ovary (CHO) cells.

**Table 1:** Comparison of the cytoplasm conductivity of various cells in different physiological states.

Cell line	Normal	Altered	Condition
Jurkat cells	0.9–0.7 S/m	0.2–0.1 S/m	Apoptotic^4^
Human Oral Keratinocytes	0.7 S/m	0.3 S/m	Oral squamous cell carcinomas^49^
HN5 cells	0.5 S/m	0.18 S/m	Cancer treated (With Cisplatin + Iressa)^9^
Stem cells	0.49 S/m	0.84 S/m	Stem cell differentiation^10^
MCF-7 cell line (Human breast)	0.23 S/m	0.4–0.14 S/m	Multidrug resistance derivatives^50^
Chinese Hamster Ovary cells	0.42 S/m	0.06 S/m	Apoptotic^51^
Chinese Hamster Ovary cells	0.4 S/m	0.32 S/m	Stationary phase of Fed-batch culture^52^
Chinese Hamster Ovary cells	0.37 S/m	0.45 S/m	Decline phase of Batch culture^52^
Chinese Hamster Ovary cells	0.42 S/m	0.27 S/m	Inhibition of mitochondria ATP production^3^
Multidrug-resistant leukaemic cells(K562AR)	0.5 S/m	0.25 S/m	Cl^−^ channel blocked with NPPB^53^
Multidrug-resistant leukaemic cells(K562AR)	0.5 S/m	0.34 S/m	K^+^ channel blocked with quinine^53^
Multidrug-resistant leukaemic cells(K562AR)	0.5 S/m	0.41 S/m	Ca^+^ channel blocked with verapamil^53^
Human chronic myelogenous leukaemia cells	0.25 S/m	0.45 S/m	4 hours incubation with Staurosporine^5^
Jurkat cells	0.5 S/m	0.9 S/m	Low conductive buffer (0.06 S/m)^54^
Chondrocytes	0.4 S/m	0.55 S/m	Low conductive buffer (0.06 S/m)^54^

Ion transport is commonly modelled using a set of nonlinear equations governing the cell volume, transmembrane potential, and internal and external ion concentrations. The model has been successful in estimating the dynamic ion transport in various cell types^12–18^. Among the reported quantitative studies, estimation of ion concentrations have been verified with experimental measurements on rat renal collecting duct (OMCD) principal cells^16,17^. In addition, quantitative models have been developed without experimental verification for human red blood cells and reticulocytes^19,20^, guinea-pig cardiomyocytes^21^, and frog skeletal muscle^13,15^. A complete model of ion fluxes and cytoplasm conductivity for CHO cells under varying cell physiology does not exist. In order to develop a quantitative model for a specific cell type, information about the density of ion channels and pumps and ion fluxes through the channels and Na^+^/K^+^ ATPase pumps are required. These parameters are known to vary from one cell type to another^15,16,22^ and have not been previously determined for CHO cells. There are various techniques to study ion channels and pumps activity such as binding assays, electrophysiological assays, flux-based assays and fluorescence-based assays^23–26^. Flux-based assays are common to study changes in ion channels activity by measuring cell membrane ion flux using isotopes or tracer elements^26^.

In this study, we develop a predictive model of cytoplasm conductivity for CHO cells. We determine the density of pumps and channels for CHO by measuring ion fluxes. To determine ion fluxes through channels and pumps in CHO cells we employ a flux-based assay with a Rb^+^ as a tracer element. To separate the ion fluxes through the Na^+^/K^+^ ATPase pumps and channels, Rb^+^ and K^+^ free buffers are used. Employing the obtained parameters in the ion transport model, we predict temporal changes in the cytoplasm conductivity of CHO cells. We verify the model predictions by comparing its results with measured cytoplasm conductivity of healthy and pump-inhibited CHO cells using Ouabain^27,28^. Measurement of cytoplasm conductivity is performed at a single cell level using a dielectrophoresis (DEP) cytometer^29–31^. The developed model provide an insight on how cells ionic balance vary in different environmental conditions. The model is applicable to other mammalian cell lines with proper parameter adjustments.

## Results and Discussion

### Mathematical Model of Cell Ion Transport

**Figure 1:**
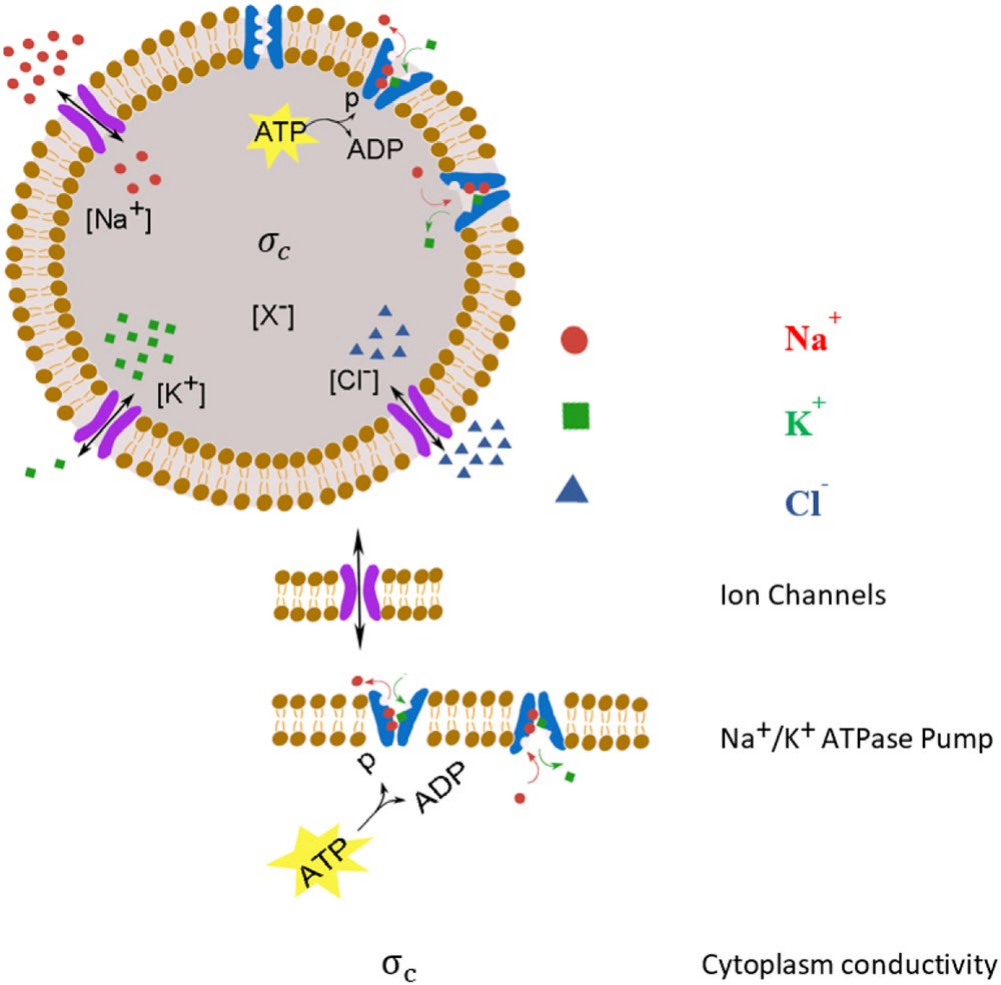
The model cell in normal condition. Its membrane contains three types of channels, Cl^−^, K^+^, and Na^+^ which mediate passive movement of these ions and Na^+^/K^+^ ATPase pumps which produce efflux Na^+^ ions and influx K^+^ ions with a 3:2 ratio. [X^−^] are the membrane impermeable anion concentration.

The mathematical model employed in this work is based on approaches proposed in literature^14–18^. To model the temporal ion transport across the membrane, we consider passive channels for sodium, potassium, and chloride and an active pathway via Na^+^/K^+^ ATPase pumps (Figure 1). Ion fluxes through the other co-transporters such as KCC (K^+^-Cl^−^) and NKCC (Na^+^-K^+^-Cl^−^) are assumed to be negligible according to experimental data shown in^16^. In the model, ions and water transport depend on the number of passive channels and Na^+^/K^+^ ATPase pumps, which are assumed to remain constant during the time period of the simulation.

The set of equations governing the dynamics of the number of intracellular moles of ions (*n*_*Na*_, *n*_*K*_, *n*_*Cl*_) and cell volume due to the movement of water and ions are^16^,

1 $$\frac{d{n}_{Na}}{dt}=A(\,-\,3\,{J}_{p}+{J}_{Na}),$$

2 $$\frac{d{n}_{K}}{dt}=A(2\,{J}_{p}+{J}_{K}),$$

3 $$\frac{d{n}_{Cl}}{dt}=A({J}_{Cl}),$$

4 $$\frac{d{V}_{C}}{dt}=A{V}_{w}\,{P}_{w}(\,\frac{{n}_{Na}+{n}_{K}+{n}_{Cl}+{n}_{X}}{{V}_{c}}-{\Pi }_{e}\,),$$

where, J_i_ (i = K, Na, Cl) are the inward ion fluxes, J_p_ is the Na^+^/K^+^ ATPase pump flux, A, V_w_, P_w_ and Π_*e*_ are cell surface area, partial molar volume of water, membrane osmotic water permeability, and extracellular osmolarity respectively and defined in Table 2. To satisfy osmotic balance and electroneutrality $(\,\frac{{n}_{Na}+{n}_{K}+{n}_{Cl}+{n}_{X}}{{V}_{c}}={\Pi }_{e},{n}_{Cl}+{Z}_{X}\,{n}_{X}={n}_{Na}+{n}_{K})$ the cell is assumed to contain a fixed number of membrane impermeable anions, n_x_, with the mean charge valence of Z_X_. These two equations are employed to estimate Z_x_ and n_x_. The estimated value of Z_X_ is equal to −1.2 which is in the range reported in literature^14–16^.

**Table 2:** Parameters, associated symbols, and their values employed for CHO cell model.

Parameter	Symbol	Value (unit)	Reference
Extracellular osmolarity	*Π*_*e*_	300 mM	measured
Intracellular ATP concentration	[*ATP*]_*i*_	5 × 10^−6^ mol/cm^3^	^16, 35^
Intracellular ADP concentration	[*ADP*]_*i*_	6 × 10^−8^ mol/cm^3^	^16, 35^
Intracellular inorganic phosphate	[*P*_*i*_]_*i*_	4.9 × 10^−6^ mol/cm^3^	^16, 35^
Ratio of permeabilities	*P*_*Na*_: *P*_*K*_: *P*_*Cl*_	1:1:5.7	Calc.^36^
Membrane *Na*^+^ permeability	*P*_*Na*_	5.6 × 10^−8^ cm/s	Calc.
Membrane *K*^+^ permeability	*P*_*K*_	5.6 × 10^−8^ cm/s	Exp.
Membrane *Cl*^−^ permeability	*P*_*Cl*_	3.2 × 10^−7^ cm/s	Calc.
Membrane osmotic water permeability	*P*_*w*_	0.0012 cm/s	^55^
Partial molar volume of water	*V*_*w*_	18 cm^3^/mol	^16^
*Na*^+^/*K*^+^- ATPase pump density	N	2.56 × 10^−11^ mol/cm^2^	Exp.
Mean organic osmolyte valence	*z*_*X*_	−1.2	Calc.
Cell surface area	*A*	5.3 × 10^−6^ cm^2^	Exp.
Intracellular amount of *X*^−^	*n*_*X*_	7.5 × 10^−14^ mol	Calc.

The modified Goldman equations by Hodgkin and Katz are used to model the passive ion movement through the channels (J_i_)^32–34^,

5 $${J}_{Na}={P}_{Na}\varepsilon (u)[{[N{a}^{+}]}_{e}\,{\exp }(-\frac{u}{2})-{[N{a}^{+}]}_{i}\,{\exp }(\frac{u}{2})],$$

6 $${J}_{K}={P}_{K}\varepsilon (u)[{[{K}^{+}]}_{e}\,{\exp }(-\frac{u}{2})-{[{K}^{+}]}_{i}\,{\exp }(\frac{u}{2})],$$

7 $${J}_{Cl}={P}_{Cl}\,\varepsilon (u)[{[C{l}^{-}]}_{e}\,{\exp }(\frac{u}{2})-{[C{l}^{-}]}_{i}\,{\exp }(-\frac{u}{2})],$$

where, *u* = *F E*_*m*_/*RT*, *ε*(*u*) *= u*/(exp (*u*/2) − exp (−*u*/2)), *P*_*Na*_, *P*_*K*_, and *P*_*Cl*_ *are* membrane ion permeabilities and F, R and T are Faraday’s constant, gas constant and absolute temperature, respectively.

The Na^+^/K^+^ ATPase pump flux is derived from a six-stage sequential kinetic model of Na^+^/K^+^ ATPase pump activity reported in^35^ as,

8 $${J}_{p}=\frac{N}{\Sigma }(\alpha -\beta ),$$

where, α is a function of the forward rate constants, *β* is a function of the backward rate constants, N is the Na^+^/K^+^ ATPase pump density, and Σ is a function of all the rate constants and ligand concentrations. In this work, the constant parameters of the Na^+^/K^+^ ATPase pumps reported in^14^ are used.

To estimate the membrane potential, E_m_, a stationary solution of the electroneutral condition is employed and defined as^14^,

9 $$-{J}_{p}+{J}_{Na}+{J}_{K}-{J}_{Cl}=0.$$

Substituting Eqs 5–8 in Eq. 9, an expression for E_m_ is derived as,

10 $${{\rm{E}}}_{{\rm{m}}}=\frac{{\rm{RT}}}{{\rm{F}}}ln(\frac{({{\rm{P}}}_{{\rm{K}}}{[{{\rm{K}}}^{+}]}_{{\rm{e}}}+{{\rm{P}}}_{{\rm{Na}}}{[{{\rm{Na}}}^{+}]}_{{\rm{e}}}+{{\rm{P}}}_{{\rm{Cl}}}{[{{\rm{Cl}}}^{-}]}_{{\rm{i}}})\,\varepsilon (u)+\frac{N}{\Sigma }\beta \,\exp (\frac{u}{2})\,}{({{\rm{P}}}_{{\rm{K}}}{[{{\rm{K}}}^{+}]}_{{\rm{i}}}+{{\rm{P}}}_{{\rm{Na}}}{[{{\rm{Na}}}^{+}]}_{{\rm{i}}}+{{\rm{P}}}_{{\rm{Cl}}}{[{{\rm{Cl}}}^{-}]}_{{\rm{e}}})\,\varepsilon (u)+\frac{N}{\Sigma }\alpha \,\exp (-\frac{u}{2})}).$$

The fixed and variable parameters used in the model for CHO cells, their values and associated symbols are listed in Table 2. A fourth order Runge-Kutta method is used to numerically solve the differential equations.

To verify the mathematical model described here, we simulate the steady state internal ion concentrations and membrane potential of two cell lines (OMCD^16^ and skeletal muscle cell^15^). We compared our simulation results with the results previously reported in literature^15,16^. Table 3 shows the comparison results. In our simulations parameters reported in literature^15,16^ were employed for OMCD and skeletal muscle cells.

**Table 3:** Membrane potential and ion concentrations of OMCD principal cells and skeletal muscle cells.

Physiological characteristics	OMCD*	OMCD^16^	Skeletal muscle*	Skeletal muscle^15^
E_m_ (mV)	−36	−37	−86	−88
[Na^+^]_i_ (mM)	36	37.3	15	21
[K^+^]_i_ (mM)	123	124	124	121
[Cl^−^]_i_ (mM)	36	32.2	5	3.8
[X^−^]_i_ (mM)	85	86	86	84

The columns marked with *show the results using the model described in this study.

### Measurement of ion fluxes through channels and Na^+^/K^+^ ATPase pumps

In this work, we used rubidium (Rb^+^) as a tracer of potassium^27,36^. We measured K^+^ and Rb^+^ concentrations inside adherent CHO cells in three experiments with media containing either K^+^ or Rb^+^ using inductively coupled plasma optical emission spectroscopy (ICP-OES, Varian 725-ES, Agilent, Australia). The obtained concentrations and their rate of change over time were employed to calculate K^+^ and Rb^+^ fluxes across the membrane channels and Na^+^/K^+^ ATPase pumps. The cell radius and number of cells required for flux calculations were determined by optical imaging using a Cedex XS cell analyzer (Innovatice, Germany).

**Figure 2:**
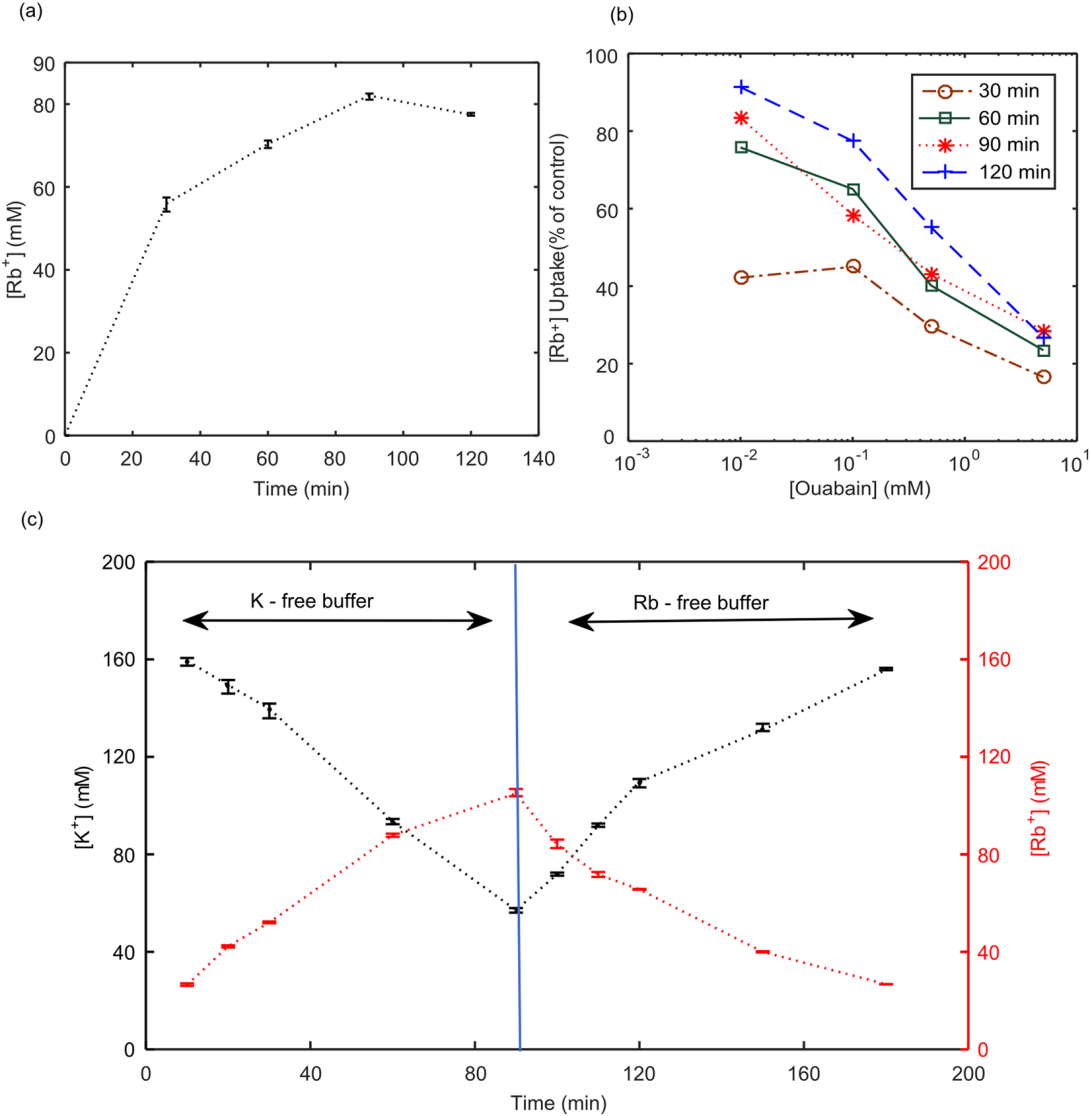
(**a**) Rubidium uptake by adherent CHO cells in a K^+^-free buffer over a period of two hours. (**b**) Percentage of Rubidium uptake by adherent CHO cells treated with various concentrations of Ouabain with respect to untreated cells in (**a**). Ouabain inhibits the Na^+^/K^+^ ATPase pumps. (**c**) Potassium and Rubidium concentration inside CHO cells in a K^+^-free buffer (0–90 min) and subsequently in a Rb^+^-free buffer (90–180 min). The error bars represent the minimum and maximum values of the ion concentrations for three repeated measurements reported by ICP-OES.

The cell radius was measured four times using a Trypan Blue exclusion assay. The uncertainty of measurements is ±4% on the radius and ±8% on the surface area of the cell. The average cell diameter was measured to be 13 μm and there were approximately 6.8 × 10^6^ cells in each T25 cm^2^ flask. In the first experiment, CHO cells were placed in a K^+^-free buffer containing RbCl (5.4 mM). Figure 2(a) shows the change in the intracellular concentration of Rb^+^ over a 2-hour period. In this case, Rb^+^ transport into cells occurs through both channels and the Na^+^/K^+^ ATPase pumps. Rb^+^ concentration inside the cells reaches a plateau (approximately 80 mM) after 90 minutes. In this case, the total flux through channels and the Na^+^/K^+^ ATPase pumps is J_Rb-tot_ = 4.6 × 10^−12^ mol.cm^−2^.s^−1^ In the second experiment, cells were placed in the same K^+^-free buffer containing RbCl (as in previous experiment) and Ouabain was added to inhibit the Na^+^/K^+^ ATPase pumps activity. Figure 2(b) shows the effect of different concentrations of Ouabain on the uptake of Rb^+^. It is evident that inhibition of Rb^+^ uptake through the Na^+^/K^+^ ATPase pumps (reduction of 76% as compared to no Ouabain in Figure 2(a)) is achieved after 2-hour incubation with 5 mM of Ouabain. This is in agreement with the results reported in literature^27^. In this case, Rb^+^ is transported into the cells solely through the channels and the measured result obtains the Rb^+^ flux through the channels J_Rb_ = 1.1 × 10^−12^ mol.cm^−2^.s^−1^. Using the Rb^+^ flux through the channels, J_Rb_, and considering the total Rb^+^ flux through channels and the Na^+^/K^+^ ATPase pumps, J_Rb-tot_, the Rb^+^ flux through the Na^+^/K^+^ ATPase pumps is calculated as, J_Rb-p_ = 3.5 × 10^−12^ mol.cm^−2^.s^−1^. In this case, some of the channels are contributing to efflux of potassium. In the third experiment cells were initially incubated in the K^+^-free buffer containing RbCl for 90 minutes (similar to the first experiment) and then washed and incubated in a Rb^+^-free buffer containing KCl for another 90 minutes. The measured potassium and rubidium content of cells over the 180 minutes is shown in Figure 2(c). The error bars represent the minimum and maximum values of the ion concentrations for three repeated measurements reported by ICP-OES for each time interval. Over the initial 90 minutes (in the K^+^-free buffer containing Rb^+^), efflux of K^+^ occurs through the channels and the potassium concentration inside the cells decreases over time, as shown in Figure 2(c). This result obtains the K^+^ flux through the channels, J_K_ = −5.9 × 10^−12^ mol.cm^−2^.s^−1^. Considering the obtained Rb^+^ and K^+^ fluxes, the total flux through the channels is 7 × 10^−12^ mol.cm^−2^.s^−1^. The total flux through the channels is calculated by adding the magnitude of J_K_ and J_Rb_ (regardless of direction) as in both transport mechanisms the passive channels are involved. Over the same period, Rb^+^ enters the cells through both channels and Na^+^/K^+^ ATPase pumps. During the next 90 minutes where the cells were placed in the Rb^+^-free buffer containing KCl, efflux of Rb^+^ occurs only through the channels and influx of K^+^ takes place through the channels and Na^+^/K^+^ ATPase pumps. The Rb^+^ flux calculated from this experiment is J_Rb_ = −5.8 × 10^−12^ mol.cm^−2^.s^−1^. Considering the total flux through the channels, we conclude that, potassium flux through the channels is 1.2 × 10^−12^ mol.cm^−2^.s^−1^ from 90 to 180 minute. The total K^+^ flux through the channels and the Na^+^/K^+^ ATPase pumps is calculated as, J_K-tot_ = 4.7 × 10^−12^ mol.cm^−2^.s^−1^. From this experiment K^+^ flux through the Na^+^/K^+^ ATPase pumps is calculated as, J_K−p_ = 3.5 × 10^−12^ mol.cm^−2^.s^−1^. Note that by separate measurements of K^+^ and Rb^+^, similar values were obtained for J_K−p_ and J_Rb−p_. This is expected as potassium and rubidium employ the same Na^+^/K^+^ ATPase pumps for active transportation across the membrane. The uncertainties in measured ion concentrations (Figure 2(c)) and radius cause maximum 6% and 4% deviation in the calculated ion fluxes, respectively, which is negligible. Note that a large number of parameters are used in the calculations. However the sensitivity of the calculations to each of the parameters is not uniform due in part to the non-linear nature of the calculations. The sensitivity of the calculations to these parameters are reported in Supplementary Information. The obtained fluxes along with Eqs 1–10 were employed to calculate the membrane ion permeabilities, P_Na_, P_K_, and P_cl_, and the Na^+^/K^+^ ATPase pump density, N, for CHO cells. The values are listed in Table 2.

### Simulation results of cell volume, membrane potential and intracellular ion concentrations

**Figure 3:**
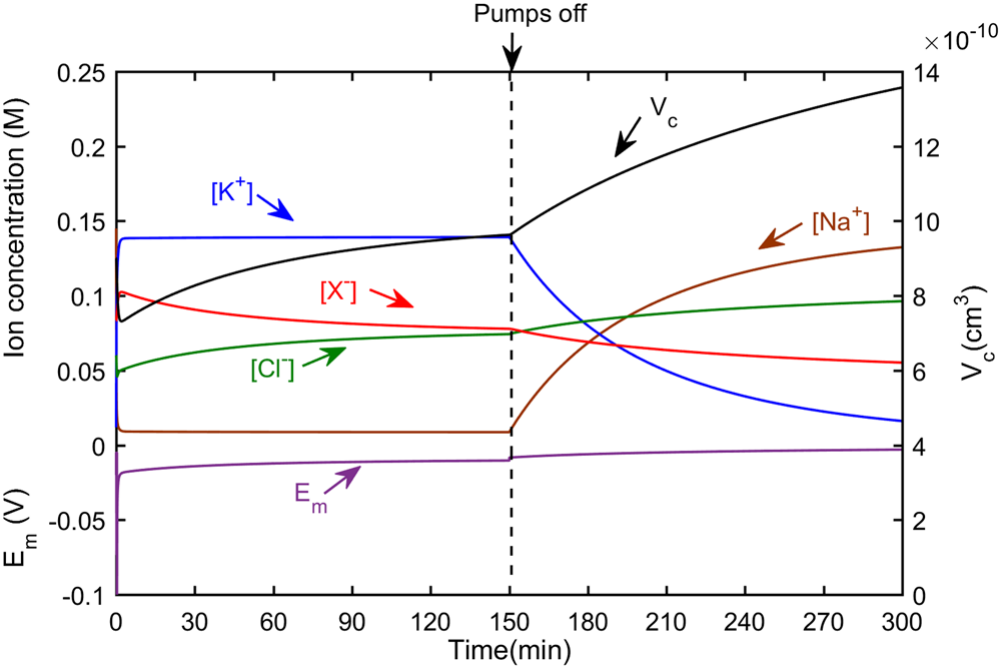
Simulation results of ion concentrations, membrane potential and cell volume for healthy and pump inhibited CHO cells in 1.7 S/m medium. The model is initiated with intracellular ion concentrations close to equilibrium with extracellular fluid except the chloride concentration which is lower due to the intracellular organic anions ([Na^+^]_i_ = 145 mM, [K^+^]_i_ = 12 mM, [Cl^−^]_i_ = 60 mM, [X^−^]_i_ = 83 mM). V_c_ is initially defined as 9 × 10^−10^ cm^3^. The parameters such as ion permeabilities and Na^+^/K^+^ ATPase pump density are as specified in Table 2. Ion concentrations at steady state are [Na^+^]_i_ = 11 mM, [K^+^]_i_ = 145 mM, [Cl^−^]_i_ = 70 mM and [X^−^]_i_ = 74 mM. At the point marked with dash line, after 150 minutes, the Na^+^/K^+^ ATPase pump density is reduced to zero, to simulate total Na^+^/K^+^ ATPase pump inhibition. At this time, the model is initiated with variables derived from the results of the first 150 minutes, marked with dash line, and therefore all variables are initially stable. After pump inhibition, there is a gradual depolarization as [K^+^]_i_ and [Na^+^]_i_ begin to equilibrate with the extracellular fluid. This depolarization allows [Cl^−^]_i_ influx and thus volume increases.

Biological cells maintain a stable cell volume, membrane potential and intracellular ion concentrations in a healthy state when the Na^+^/K^+^ ATPase pumps and channels function properly. The model described here (see Mathematical Model of Cell Ion Transport and Cytoplasm Conductivity section) was employed to simulate the steady state of a normally functioning cell with extracellular concentrations set similar to those of regular growth media (1.7 S/m). In addition, the effect of various perturbations such as shutting down the pumps or changing the medium conductivity on the cell ion concentrations. Figure 3 summarizes the simulation results for membrane potential, cell volume and ion concentrations within a cell and after shutting down the Na^+^/K^+^ ATPase pumps. The model is initiated with intracellular ion concentrations close to equilibrium with extracellular fluid except the chloride concentration which is lower due to the intracellular organic anions ([Na^+^]_i_ = 145 mM, [K^+^]_i_ = 12 mM, [Cl^-^]_i_ = 60 mM, [X^−^]_i_ = 83 mM, E_m_ = −20 mV and V_c_ = 9 × 10^−10^ cm^3^). Channels and pumps parameters obtained for CHO in “Measurement of ion fluxes through channels and Na^+^/K^+^ ATPase pumps” section were employed in simulations. The model parameters are reported in Table 2. Note that the ratio of potassium to sodium passive channels is 1:1 for CHO cells^36^ and their steady state membrane potential is −10 mV^37^. These are different from most mammalian cells for which the ratio of potassium to sodium is 1:50 and their membrane potential is −88 mV^15^. The simulation results of membrane ion permeabilities, Na^+^/K^+^ ATPase pumps density, the steady state ion concentrations and membrane potential of CHO cells in comparison with OMCD and skeletal muscle cells are presented in Table 4. It shows how these parameters vary for different cell lines requiring proper characterization for accurate modelling. Figure 3 also shows that by inhibiting the Na^+^/K^+^ ATPase pumps activity, the cells ion contents begin to equilibrate with the extracellular fluid ([Na^+^]_e_ = 145 mM, [K^+^]_e_ = 5 mM, [Cl^−^]_e_ = 110 mM and other compounds). It is in agreement with the results of pump inhibition reported in literature^15,38^ which shows [K^+^]_i_ and [Na^+^]_i_ begin to equilibrate with the extracellular fluid after the pump inhibition. This allows [Cl^−^] influx and thus volume increases^15,38^.

**Table 4:** Comparison of physiological characteristics.

Physiological characteristics	CHO cell (This study)	OMCD cells^16^	Skeletal muscle cells^15^
P_Na_ (cm/s)	5.6 × 10^−8^	3.2 × 10^−6^	8 × 10^−10^
P_K_ (cm/s)	5.6 × 10^−8^	1 × 10^−5^	4 × 10^−8^
P_Cl_ (cm/s)	3.2 × 10^−7^	3 × 10^−6^	1.2 × 10^−7^
N (mol/cm^2^)	2.56 × 10^−11^	3.35 × 10^−12^	5 × 10^−12^
E_m_ (mV)	−12 ± 1*	−37	−88
[Na^+^]_i_ (mM)	11 ± 1*	37.3	21
[K^+^]_i_ (mM)	145 ± 6*	124	121
[Cl^−^]_i_ (mM)	70 ± 2*	32.2	3.8

^•^The reported uncertainties here is due to the uncertainty in measured radius.

### Estimation of cytoplasm conductivity from simulation model

The simulated ion concentrations shown in Figure 3 are used to estimate the cytoplasm conductivity (σ_c_) of CHO cells. In order to calculate the conductivity from ion concentrations, a simplified version of the Kohlraush law and limiting molar conductivity of ions in water has been used^39^, given as,

11 $${{\rm{\sigma }}}_{c}=\mu \{{}_{{\rm{Na}}}[{{\rm{Na}}}^{+}]{\rm{i}}+{{\rm{\lambda }}}_{{\rm{K}}}[{{\rm{K}}}^{+}]{\rm{i}}+{{\rm{\lambda }}}_{{\rm{Cl}}}[{{\rm{Cl}}}^{-}]{\rm{i}}\}.$$

Here *λ*_*i*_ (i = Na, K, Cl) is the limiting molar conductivity of ion i in water^40^, [i^+^] is the concentration of ion i and μ is the mobility factor. The mobility in the cytoplasm is 0.25-0.35^40^ and is less than 1. It can be attributed to the presence of organelles, proteins and other molecules in cytoplasm reducing the space available for the ions to move as well as other scattering influences. Therefore, the effective mobility in the cytosol is 3-4 times lower than estimated from the limiting molar conductivity^39,41,42^.

### Comparison of model simulation and experimental results of the effect of Na^+^/K^+^ pump inhibition

**Figure 4:**
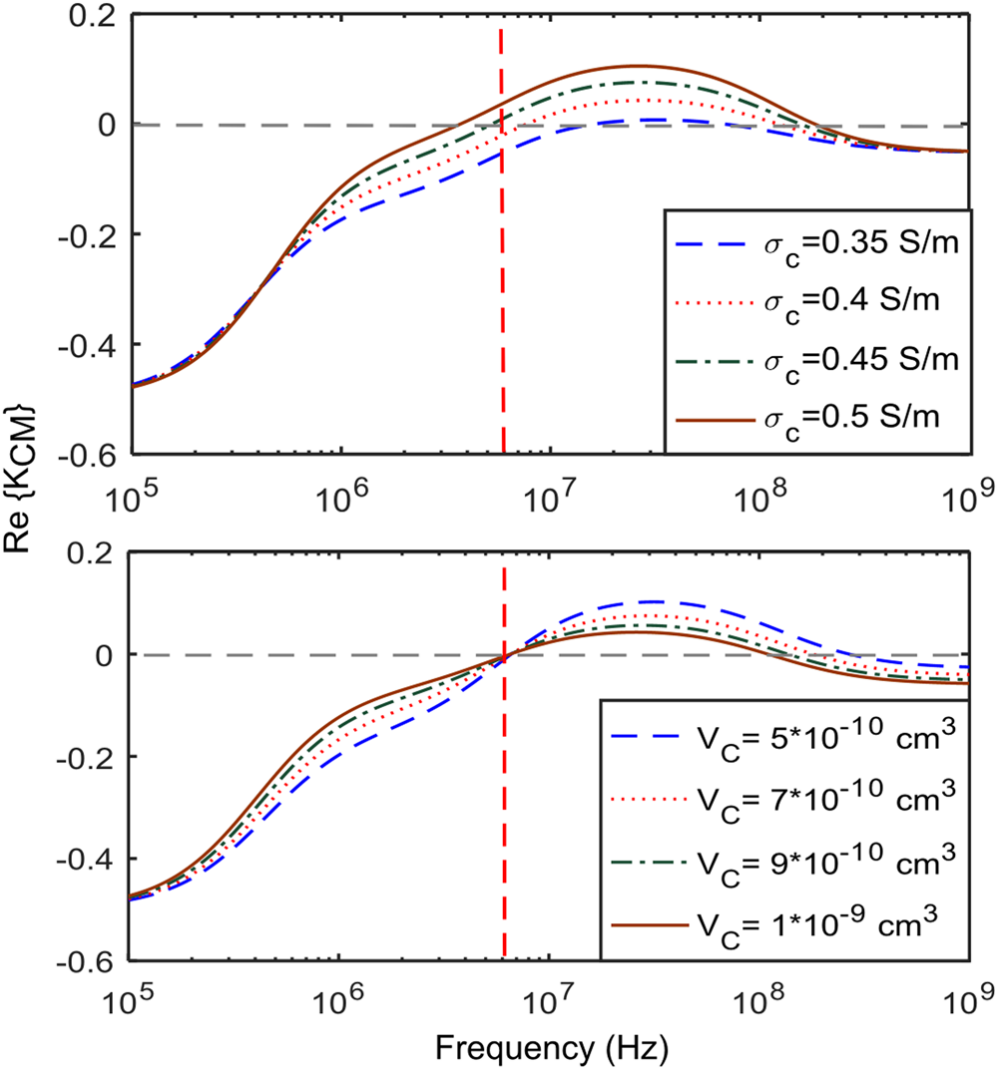
Simulated spectrum of the real part of the Claussius-Mossotti factor (Re {K_CM_}) for a mammalian cell (Chinese hamster ovary (CHO)), with parameters from^29^. (**a**) Cytoplasm conductivity varies from 0.35–0.5 S/m and medium conductivity is 0.42 S/m. (**b**) Cell volume varies from 5 × 10^−10^–10 × 10^−10^ cm^3^ and medium conductivity is 0.42 S/m.

There has been studies showing that cytoplasm conductivity plays an important role in different biological processes such as apoptosis^4–6^, progression of cancer^7–9^, differentiation of stem cells^10^, separation of healthy and tumor cells^11^. One aim of this work is to provide a link between physiological changes and cytoplasm conductivity of CHO cells. In this work, Dielectrophoresis has been chosen as the method to monitor the cytoplasm conductivity of CHO cells. This can be achieved with a time resolution of a few minutes. Dielectrophoresis is the translation of a polarizable particle in a non-uniform electric field. The time averaged DEP force exerted on a cell is given by^43^

12 $${\overrightarrow{F}}_{DEP}=1.5\,{{\rm{\varepsilon }}}_{m}\,{V}_{c}\,Re\{{K}_{CM}\}.\overrightarrow{\nabla }|{\bar{E}}_{rms}^{DEP}(r){|}^{2},$$

where, V_c_ is the cell volume, $\bar{E}\,$ is the non-uniform electric field at the position of the cell, and K_CM_ is the Claussius-Mossotti factor, expressed as,

13 $${K}_{CM}=\frac{{\tilde{\varepsilon }}_{p}-{\tilde{\varepsilon }}_{m}}{{\tilde{\varepsilon }}_{p}+2{\tilde{\varepsilon }}_{m}},$$

where, ${\tilde{\varepsilon }}_{p}$ and ${\tilde{\varepsilon }}_{m}$ are the complex permittivity of the cell and medium, respectively, defined as ε = ε_0_ε_r_ + σ/jω, with ω being the angular frequency of the electric field. It should be noted that, through K_CM_, the DEP response is dependent on the difference in the complex permittivity of the cell and medium. The amount of deflection and its direction due to the DEP force is directly related to the magnitude and sign of Re {K_CM_}. Re {K_CM_} is depicted in Figure 4 for a typical healthy CHO cell in a medium with conductivity 0.42 S/m. The dielectric properties of different compartments of cells affect the Re{K_CM_} spectrum in different frequency ranges. For example, Re{K_CM_} is dominantly affected by the cytoplasm conductivity, and subsequently the ionic composition of the cell at frequency of 6 MHz^3^, shown in Figure 4(a). This operating point is chosen, as it is least sensitive to other factors such as cell size, shown in Figure 4(b). In CHO cells the equilibrium cytoplasm conductivity is approximately 0.46 S/m in a medium with conductivity 0.42 S/m. This is obtained experimentally as Re{K_CM_} ≈ 0 resulting in a near zero DEP force on cells.

**Figure 5:**
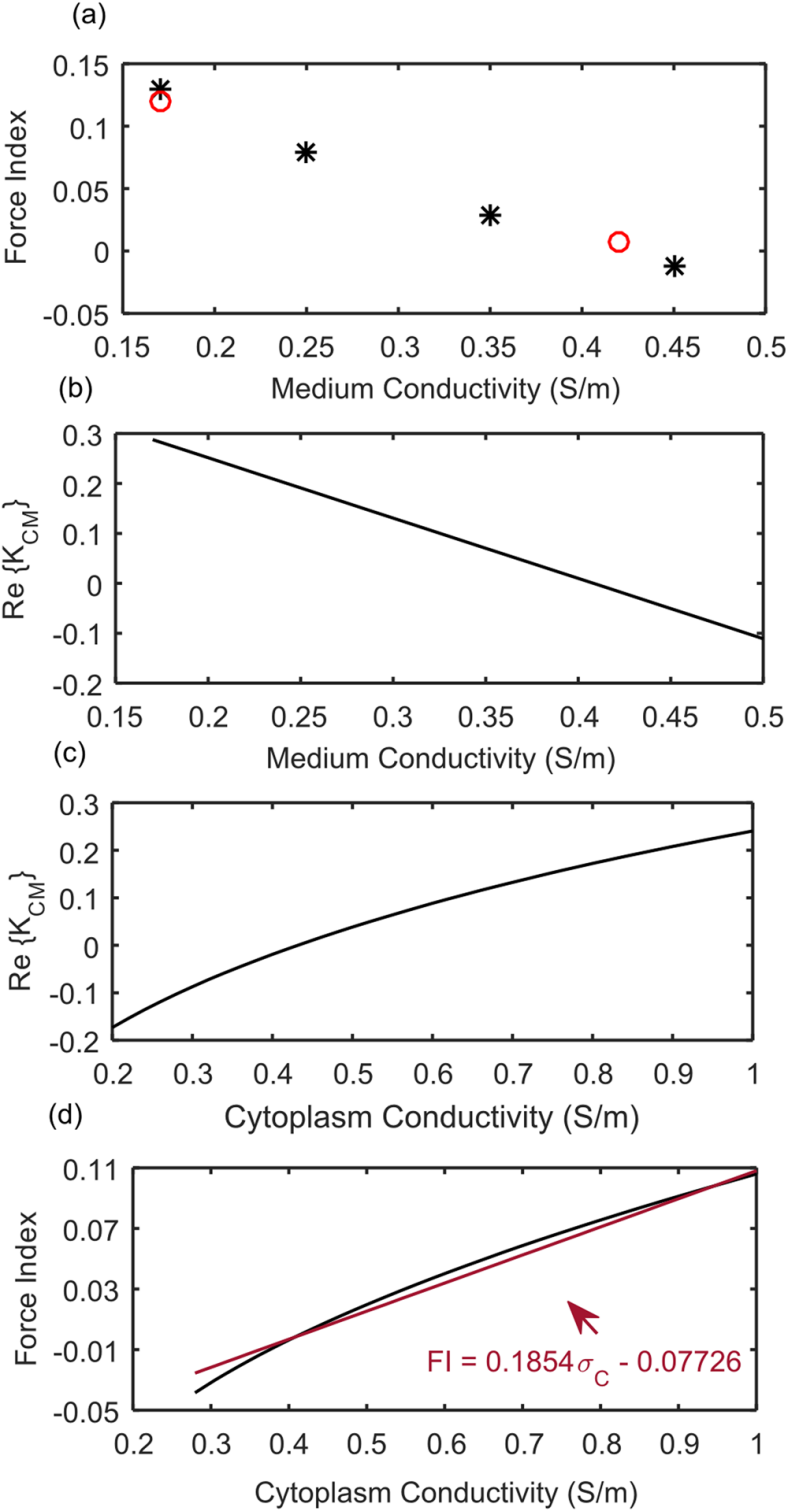
(**a**) Mean Force Index for 0.17–0.45 S/m medium conductivities. Black stars indicate the obtained results in our previous work^3^, red circles indicate the results obtained in this work. (**b**) The real part of the K_CM_ calculated using a double shell model versus the medium conductivity. (**c**) The real part of the K_CM_ calculated using a double shell model versus the cytoplasm conductivity. (**d**)The linear relation between force index and cytoplasm conductivity using (**a**–**c**) graphs.

The detail of the DEP cytometer used in this work to monitor the cytoplasm conductivity of the cells is described in Supplementary Information. The DEP cytometer measures a parameter called force index which is related to the cells displacement due to an applied DEP force. To map the obtained experimental results by DEP cytometer (Force Index) to the Re {K_CM_} and subsequently cell cytoplasm conductivity (σ_C_)^29^, we followed the same procedure explained elsewhere^3^. In the prior work, first the force index of CHO cells were measured at 6 MHz frequency in medium with different conductivity shown in Figure 5(a) ^3^. Then this result was used along with the simulation result of the Re {K_CM_} for different medium conductivities and cytoplasm conductivities (see Figure 5(b,c)) to relate the measured force indices to the Re {K_CM_} and subsequently the cell cytoplasm conductivity. A linear relationship between force index and cytoplasm conductivity was established for our experimental condition (cell’s velocity, medium conductivity, etc.) and shown in Figure 5(d).

**Figure 6:**
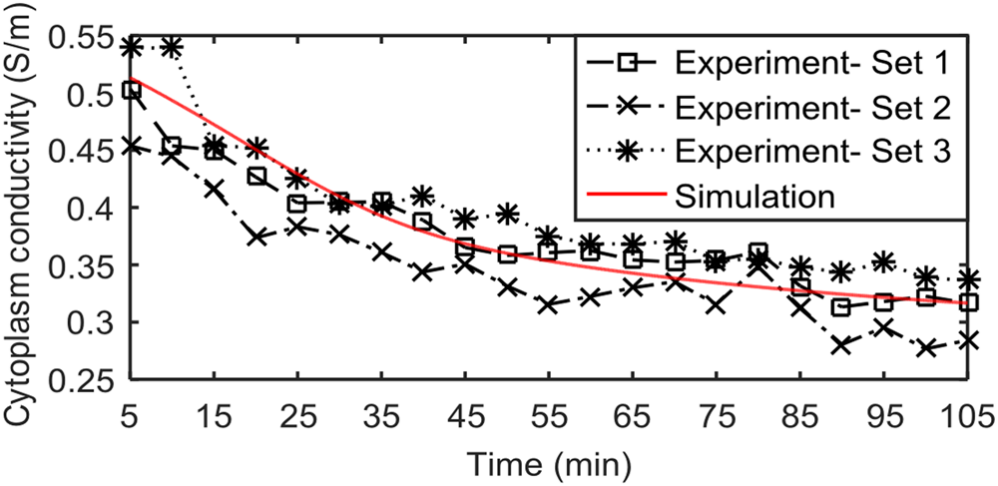
Simulation and experimental results of pump inhibited CHO cells using 5 mM Ouabain. The marked lines show the estimated cytoplasm conductivity using the experimental results of three DEP measurements for 105 min. Each marker represents the average for 5 min intervals (100–150 cells). The solid line represents the simulation results of the pump inhibited CHO cells. To inhibit the pumps, pump density is set to zero.

We simulated and experimentally measured the temporal change in the cytoplasm conductivity of Na^+^/K^+^ ATPase pump inhibited CHO cells using our model and the DEP cytometer. In the simulation model, pump inhibition was performed by setting the density of the pumps to zero for a cell at its equilibrium state in an extracellular fluid with the conductivity set to 0.42 S/m ([Na^+^]_e_ = 55.3 mM, [K^+^]_e_ = 0.8 mM, [Cl^−^]_e_ = 25.2 mM and other compounds). In experimental measurement 5 mM Ouabain was employed to inhibit Na^+^/K^+^ ATPase pumps (See Figure 2(b)). The DEP response of cells was measured in a medium with conductivity 0.42 S/m. Approximately 2500 cells were measured over a period of 105 minutes after the inhibition of the Na^+^/K^+^ ATPase pumps. The results were averaged over five minute periods (~100-150 cells in a five-min window). Figure 6 shows the simulation and experimental results of three independent experiments for the temporal change of cytoplasm conductivity in pump inhibited CHO cells. Experimental and simulation results show that there is a decline in cytoplasm conductivity of CHO cells when the pumps are inhibited. The reason is that when the pumps are shutdown, ions passively flow in and out of the cell to reach to a new equilibrium with extracellular fluid ([Na^+^]_e_ = 55.3 mM, [K^+^]_e_ = 0.8 mM, [Cl^−^]_e_ = 25.2 mM and other compounds). K^+^ and Cl^−^ flow out of the cell while Na^+^ flows into the cell resulting in a new ionic equilibrium state. The efflux of Cl^−^ and lower limiting molar conductivity of Na^+^ compared to K^+^ lead to lower cytoplasm conductivity. With the pumps off, it might be expected that the cytoplasm ion concentrations will become equal to that of the external medium. In this case, the cytoplasm conductivity would be expected to decrease to 0.11–0.14 S/m, from Eq. 11. However, the observed conductivity under pump inhibition is much larger. We hypothesize the reason is that the immobile anions within the cytoplasm require the presence of cations to maintain electroneutrality. These cations make the cytoplasm conductivity significantly larger than 0.14 S/m. The equilibrium cytoplasm conductivity obtained in our study is 0.32 S/m.

The developed model can be employed to predict the effect of physical processes such as apoptosis on the cytoplasm conductivity. Apoptosis is a central process in microbiology and biotechnology. Previous studies have shown a dramatic decrease in cells’ cytoplasm conductivity, from 0.3-0.6 S/m to 0.1-0.2 S/m, during apoptosis^4,6^. At the same time cation concentrations have been observed to drop from 140 to 30-50 mM^4,44,45^. Our model can be used to offer some insight into the movement of ions during apoptosis and subsequent changes in the cytoplasm conductivity. Using the model, we can explore what mechanisms would lead to a drop in ion concentration of this magnitude. Intuition would lead to the assumption that increases in channels ion fluxes (imitating the increase in membrane permeability observed in apoptosis) or decreases in Na^+^/K^+^ ATPase pumps density (imitating the impairment of ATP-driven pumps during apoptosis) would lead to a drop in the ion concentration and hence the cytoplasm conductivity. However, our model simulation shows that, by complete shutdown of active pumps or one hundred fold increase in channels ion fluxes, the cytoplasm conductivity decreases from 0.54 S/m to 0.49 S/m and 0.5 S/m, respectively (data not shown). In both cases, the cation concentrations are more than a factor of two above the concentration observed in apoptotic cells. This is because in the model the immobile anions within the cytoplasm must be balanced by an equal concentration of cations to maintain the charge balance^46^. This model predicts that the drop in ion concentration required to reach the cytoplasm conductivity observed during apoptosis, cannot be reached simply by the loss of Na^+^/K^+^ ATPase pump activity or the increase in ion pore density.

## Conclusion

In this paper, we proposed a quantitative model for the temporal ion transport across the Chinese hamster ovary (CHO) cells and used it to predict the cytoplasm conductivity considering Na^+^, K^+^ and Cl^−^ passive channels and Na^+^/K^+^ ATPase pumps for active pathways. We measured potassium and rubidium content of the cells using two different buffers and performed quantitative estimation of potassium flux through the active and passive pathways separately (see K^+^ and Rb^+^ Content of the cell section). The obtained fluxes were used in the model to estimate the ion channel and pump densities. Using the described model, the cytoplasm conductivity was estimated. These estimates of cytoplasm conductivity were compared with cytoplasm conductivity measurements carried out using Dielectrophoresis cytometry. The model predicted the experimentally estimated temporal changes in cytoplasm conductivity of pump inhibited CHO cells using Ouabain by setting the density of pumps to zero. The model will also aid in relating bulk dielectric measurements used for monitoring large scale cell cultures to physiological changes within cells applicable in biopharmaceutical production.

## Methods

### Cell culture

Chinese hamster ovary (CHO) cells (CHODG44-EG2-hFc/clone 1A7), provided by Yves Durocher of the National Research Council were used in this work. CHO cells were used in two cultures, suspension and adherent. CHO cells in suspension culture were grown in 250 ml shaker flasks and incubated at 37 °C with a 10% CO_2_ overlay on a shaker platform (120 rpm). The cells were passaged every 3–4 days with a seeding density of 2 × 10^5^ cells/ml in c-CHO serum-free medium (BioGro Technologies, Winnipeg, MB). Adherent CHO cells were grown in T-75 cm^2^ flasks and incubated in Iscove’s modified Dulbecco’s medium (IMDM) at 37 °C with a 10% CO_2_ overlay. The cells were passaged every 3–4 days in a 1:6 split (aspirate cells 1 ml in 9 ml IMDM).

### Ion flux measurement using ICP-OE

In this work, in order to estimate the required parameters for the quantitative model, the ion fluxes through the channels and pumps were determined using a flux-based assay with a tracer element. Here, Rb^+^ was used as a tracer of potassium to study the flux through the potassium channels and Na^+^/K^+^ ATPase pumps^23,24,47^. In the Rb^+^ assay, cells were incubated with a buffer containing Rb^+^ for 0.5-2 hours. At specific time intervals, cells were washed to remove extracellular Rb^+^ and channel activity was determined by measuring the rubidium concentration of the cell lysate and supernatant using an ion specific tool, inductively coupled plasma optical emission spectroscopy (ICP-OES, Varian 725-ES, (Agilent, Australia)).

### Buffers

Two different buffers were used in this work to study Rb^+^ uptake and K^+^ content of the cells: K^+^-free and Rb^+^-free buffer. The K^+^-free buffer contains 15 mM HEPES, 140 mM NaCl, 5.4 mM RbCl, 1 mM MgCl_2_, 0.8 mM NaH_2_PO_4_, and 2 mM CaCl_2_, and the pH is set to 7.4 with NaOH. The Rb^+^-free buffer contains 15 mM HEPES, 140 mM NaCl, 5.4 mM KCl, 1 mM MgCl_2_, 0.8 mM NaH_2_PO_4_, and 2 mM CaCl_2_, and the pH is set to 7.4 with NaOH.

### Sample preparation for ion measurement

In order to quantify the ion fluxes through the channels and Na^+^/K^+^ ATPase pumps, a measurement was performed in two groups. Two days prior to the experiment, the cells were washed and put into 10 T25 cm^2^ flasks with IMDM culture medium. On the day of the experiment, the first group of five T25 cm^2^ flasks were removed and the samples were washed twice with K^+^-free buffer. Then the first group was incubated with K^+^-free buffer for five different time intervals. At each time interval, the cells in the flask were washed and lysed. The second group of five T25 cm^2^ flasks were incubated for 90 minutes in K^+^-free buffer. The second group of samples were then washed twice with Rb^+^-free buffer and incubated at five different time intervals in the same Rb^+^-free buffer. At each time interval, the supernatant was removed, flasks were washed and cells lysed. During lysing, the sample was prepared by discarding supernatant, washing with deionized water, lysing and filtering the cell debris. In this work, 0.15% Sodium Dodecyl Sulphate (SDS) in DI water was used to lyse the cells. The average diameter and the total number of cells were determined by optical imaging using a Cedex XS analyser (Innovatice, Germany). The mean diameter is measured to be 13 μm and the average number of the cells is 6.67 × 10^6^ cells in each of the T-25 (surface area 25 cm^2^) flasks. Inductively coupled plasma optical emission spectroscopy, ICP-OES, Varian 725-ES, (Agilent, Australia). was used to measure the ion concentration of the filtered solution. The sample volume for ICP-OES measurement was 5 ml. In order to measure Rb^+^ uptake over time, adherent CHO cells were incubated in K^+^-free buffer, containing 5.4 mM Rb^+^. In some cases, Ouabain was also added to the buffer to determine the proper Ouabain concentration for maximum Na^+^-K^+^ ATPase pump inhibition over a period of two hours. The experiment was performed for five different Ouabain concentrations: 0, 0.01, 0.1, 0.5 and 5 mM for four different time intervals. The same procedure was used to wash and lyse the cells at each time intervals.

### Sample preparation for DEP measurement

To prepare samples for DEP measurement, cells at 2 days after seeding were taken from the shaker flask, centrifuged and resuspended in a mix of low conductivity and BioGro CHO medium to a concentration 2 × 10^5^ cells/ml. The ratio of low conductivity to BioGro CHO medium was adjusted to reach the desired media conductivity 0.42 S/m. In order to prepare pump inhibited CHO cells for DEP measurement, cells were resuspended in 0.42 S/m medium containing 5 mM Ouabain. Ouabain is known to inhibit the pump activity in several minutes^48^.

## Electronic supplementary material

SupplementaryInformation

## Data Availability

The datasets generated during and/or analysed during the current study are available from the corresponding author on reasonable request.
