# Supplementary material for: Quantitative Model for Ion Transport and Cytoplasm Conductivity of Chinese Hamster Ovary Cells
Source: Sci Rep. 2018 Dec 13;8:17818. doi: 10.1038/s41598-018-36127-3 (PMC6292909; doi:10.1038/s41598-018-36127-3)
Supplement: Supplementary file 1 — SupplementaryInformation [file 41598_2018_36127_MOESM1_ESM.pdf]

Supplementary Information

Quantitative Model for Ion Transport and Cytoplasm Conductivity of Chinese Hamster Ovary Cells

Azita Fazelkhah[1], Katrin Braasch[2], Samaneh Afshar[1], Elham Salimi[1], Michael Butler[3], Greg Bridges[1], and Douglas Thomson[1,*]

[1]Department of Electrical and Computer Engineering, University of Manitoba, Winnipeg, R3T 2N2, Canada
[2]Department of Microbiology, University of Manitoba, Winnipeg, R3T 2N2, Canada
[3]National Institute for Bioprocessing Research and Training, Dublin, Ireland
*Douglas.thomson@umanitoba.ca

Dielectrophoresis cytometer. In order to determine the actuation of individual cells using a dielectrophoresis (DEP) force, a microfluidic system with differential actuating and sensing electrodes at the bottom of the channel is employed. The DEP cytometer used in this work was developed at the University of Manitoba, as shown in Figure S1 [1,2]. A schematic view of the channel with a cell flowing over sets of coplanar electrodes is shown in Figure S1(a). As the cell passes over the first pair of detection electrodes (D1) a signal with a peak value P_1 is produced. Then the cell is exposed to a DEP force as it passes over the actuation electrode (A) to which the DEP voltage is applied. Finally, as the cell passes over the second pair of detection electrodes (D2) a signal with a peak value P_2 is produced. When a DEP voltage is applied, the cell will be attracted or repelled from the higher density electric field region depending on the magnitude and sign of the Claussius Mossotti factor. The cell holding Re $\{K_{CM}\} > 0$ experiences pDEP force and is pulled toward the region with high electric field intensity which results in $P_2 > P_1$. Conversely, the one exhibiting Re $\{K_{CM}\} < 0$ experiences nDEP force and is repelled from the region with high electric field intensity which results in $P_2 < P_1$. Examples of signals recorded for CHO cells experiencing nDEP ($P_1 > P_2$), no DEP ($P_1 = P_2$), and pDEP ($P_1 < P_2$) actuations are shown in Figure S1(b).

To normalize the experimental results, a parameter, force index, FI $= \frac{P_2 - P_1}{P_2 + P_1}$ is introduced which is a ratio of the difference and sum of each cell signature (peaks) before and after the DEP actuation. When the Re $\{K_{CM}\}$ is positive, the force index is positive and when Re $\{K_{CM}\}$ is negative the force index is negative. The force index is approximately proportional to Re $\{K_{CM}\}$ when the DEP force is small.

Sensitivity of Flux Calculations to Model Parameters. There are a large number of parameters in Table 2, which are used in the model calculations. However, the sensitivity of the calculations to each of the parameters is not uniform due in part to the non-linear nature of the calculations. For example, even if water permeability (Pw) has a large uncertainty it has a minor effect on the results even if it is changed it by 50%. This is because the permeability is high and as a result the water remains in equilibrium over a wide range of values. For a 50% change in permeability the cytoplasm conductivity in Figure 6 only changes by 0.03%. Vw is derived from fundamental constant parameters and the uncertainty is negligible.

The membrane potential and sodium: potassium ratio of permeabilities for CHO cells reported in literature were used to find the ratio of the Chlorine permeability. The reference for these parameters does not explicitly state the uncertainty, but the calculations are relatively insensitive to uncertainties in the membrane potential. For example if the membrane potential increases by ± %25 (Em= -10- -15 mV) the obtained ratio for the chlorine flux will change by 18%, 25%, respectively. One of the parameters that changes as a result of this change is the flux through the Cl^- channels. The overall impact on the calculated cytoplasm conductivity in Figure 6 will be 0.9%.

The cell radius was measured four times using a Trypan Blue exclusion assay. The mean value of these measurements has been used in our calculations. This radius is in agreement with literature values for the cell radius [3,4]. The

uncertainty of measurements is ±4% on the radius and ±8% on the surface area of the cell. The impact of this radius uncertainty on the ion concentrations and the membrane potential reported in Table 4.

The membrane potassium permeability was obtained using experimentally measured potassium and rubidium content of the cell using two different buffers and the ratio of the permeabilities were used to calculate the membrane Na^+ and Cl^- permeabilities. Three sets of measurements have been done using ICP-OES and the error bars have been added to figure 2(c). Compared to the radius uncertainty, the error in ICP-OES have significantly less impact on the estimated ion fluxes as well as ion permeability values (P_{Na}, P_K, P_{Cl}) reported in table 4.

[ATP]i, [ADP]i, [Pi]i parameters were imported from other references and they have not reported the uncertainty for these parameters and we assume the uncertainty is not a big effect on the values in Table 3. The concentration of these parameters play role in the Na+/K+ pump activity and does not affect the results obtained in figure 6, since the Na+/K+ pump activity is zero in this experiment.

n_x and z_x are dependent values and have been determined by applying the osmolarity and electroneutrality equations. Considering the total intracellular positive ion concentrations ($[Na^+ + K^+]_i$~160 mM), chloride concentration at steady state ($[Cl^-]_i = [Cl^-]_e exp\left(-\frac{Em\,F}{RT}\right)$) and CHO cell volume, the amount of impermeable anions (n_X) was cestimated. Then the electroneutrality was applied to calculate the charge valence. Note that the extracellular osmolarity (Пe) is measured using an osmometer (AdvancedV Model 3300 Micro-Osmometer, Advanced Instruments Inc., Norwood, USA).

References

1. Nikolic-Jaric, M. *et al.* Differential electronic detector to monitor apoptosis using dielectrophoresis-induced translation of flowing cells (dielectrophoresis cytometry). *Biomicrofluidics* **7,** 1–15 (2013).

2. Ferrier, G. A., Romanuik, S. F., Thomson, D. J., Bridges, G. E. & Freeman, M. R. A microwave interferometric system for simultaneous actuation and detection of single biological cells. *Lab Chip* **9,** 3406–3412 (2009).

3. Cole, H., Demont, A. & Marison, I. The Application of Dielectric Spectroscopy and Biocalorimetry for the Monitoring of Biomass in Immobilized Mammalian Cell Cultures. *Processes* **3,** 384–405 (2015).

4. Ansorge, S., Esteban, G. & Schmid, G. Multifrequency permittivity measurements enable on-line monitoring of changes in intracellular conductivity due to nutrient limitations during batch cultivations of CHO cells. *Biotechnol. Prog.* **26,** 272–283 (2010).

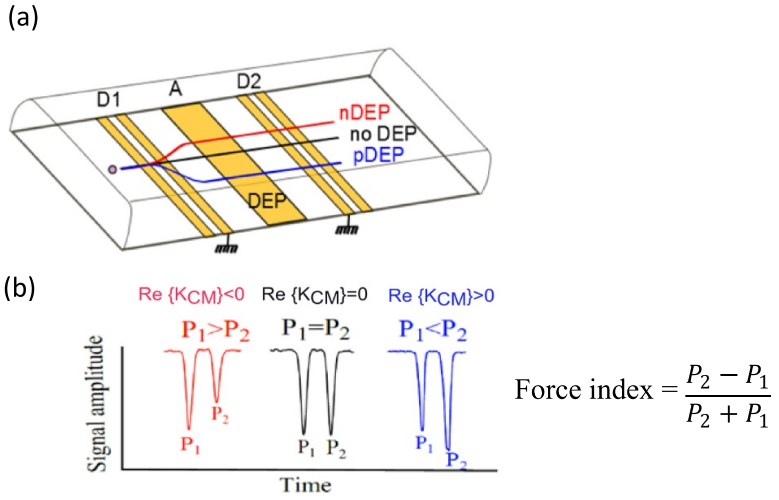

Figure. S1 (a) Schematic of the microfluidic channel. (b) Different detection signals for CHO cells experiencing nDEP force (Re $\{K_{CM}\} < 0$), no DEP actuation (Re $\{K_{CM}\} = 0$), and pDEP force (Re $\{K_{CM}\} > 0$).
